# Biomechanical characterization of human lung bullae using uniaxial tensile tests and CT-derived geometry-specific finite element analysis

**DOI:** 10.3389/fphys.2026.1818153

**Published:** 2026-08-07

**Authors:** Yan Huang, Jianan Song, Hong Fan, Guangjian Zhao, Huaiyu Wang, Ke Wu, Danya Wang, Xiaofei Wang, Zhikang Zou

**Affiliations:** 1 Department of Respiratory and Critical Care Medicine, Air Force Medical Center, PLA, Air Force Medical University, Beijing, China; 2 Laboratory for Biomechanics and Mechanobiology of Ministry of Education, Beijing Advanced Innovation Center for Biomedical Engineering, School of Biological Science and Medical Engineering, Beihang University, Beijing, China; 3 Department of Respiratory and Critical Care Medicine, Beijing Zhongguancun Hospital, Beijing, China; 4 Department of Thoracic Surgery, Air Force Medical Center, PLA, Air Force Medical University, Beijing, China; 5 Department of Medical Research, Air Force Medical Center, PLA, Air Force Medical University, Beijing, China

**Keywords:** biomechanics, bullae rupture, finite element analysis, lung bullae, uniaxial tensile tests

## Abstract

**Introduction:**

Lung bullae are air-filled spaces with thin walls located within the lungs, and they are closely associated with spontaneous pneumothorax. However, quantitative mechanical data on human bullae and subject-specific stress analyses remain sparse.

**Methods:**

We performed uniaxial tensile tests on surgically resected human tissues, including 39 bulla specimens and 34 parenchyma specimens, and fitted the nonlinear stress–strain curves using hyperelastic models. Rupture stress was calculated for specimens that ruptured within the gauge region, comprising 15 bulla specimens and 14 parenchyma specimens. We subsequently constructed 12 CT-derived geometry-specific finite element models of 12 bullae from 10 patients using preoperative CT images, with segmentation performed in Mimics and geometric smoothing performed in Geomagic. High-pressure loading of 110 cmH_2_O was simulated using shell elements for the bulla walls, and the 95th-percentile von Mises stress was used as a robust peak-stress metric.

**Results:**

A second-order Yeoh model best captured the marked strain-stiffening behavior of both tissues. The mean ultimate tensile strength was 2.318 MPa for bulla specimens and 0.390 MPa for parenchyma specimens. In the CT-derived geometry-specific finite element models, the 95th-percentile equivalent von Mises stress ranged from 50.3 to 515.8 kPa, with a median of 106.8 kPa. Elevated stresses were consistently localized to the bulla vertex or apex, defined as the most remote convex peak with the least parenchymal tethering, rather than along the equator. Under the simulated worst-case loading, no case reached the mean experimental ultimate tensile strength of bulla tissue, although one case exceeded the minimum measured bulla ultimate tensile strength of 462 kPa.

**Discussion:**

Human bulla tissue exhibits greater tensile strength than adjacent lung parenchyma but remains vulnerable to elevated local stresses generated by curvature and reduced parenchymal tethering at the apex. CT-derived geometry-specific finite element analysis using hyperelastic constitutive models provides a biomechanical explanation for the clinically observed apical predilection of bulla rupture.

## 1 Introduction

Lung bullae are air-containing spaces within emphysematous lung in which alveolar walls have coalesced, and the intervening septa are resorbed, leaving a thin fibrotic wall. Clinically, bullae can compress adjacent parenchyma (vanishing-lung syndrome), worsen dyspnea, and predispose to spontaneous pneumothorax; they also complicate ventilation and surgical decision-making (bullectomy, lung volume reduction). As imaging has improved, blebs/bullae are recognized across COPD spectra and in primary spontaneous pneumothorax (PSP), particularly at the apices (West, 1971; Mead et al., 1970; Hallifax, 2022; Park et al., 2019). In PSP, the classical “ruptured bleb/bulla” explanation has been nuanced by evidence for diffuse pleural and subpleural disease, micro-porosity, and inflammation, but focal subpleural lesions remain strongly implicated in many patients (Hallifax, 2022; Noppen, 2010; Grundy et al., 2012).

Mechanically, the lung parenchyma is a nonlinear, viscoelastic, fiber-reinforced network of elastin and collagen with strong strain-stiffening (Andrikakou et al., 2016; Polio et al., 2018; Jorba et al., 2019; Suki and Bates, 2011). Hyperelastic models—most commonly Neo-Hookean, Mooney–Rivlin, Ogden, and Yeoh forms—offer phenomenological fits of large-strain data when material constants are derived from uniaxial/biaxial tests (Andrikakou et al., 2016; Polio et al., 2018; Yeoh, 1993; Mooney, 1940; Rivlin, 1948). In emphysema, the redistribution of load from degraded elastin to collagen amplifies stiffening at higher strains, altering local stress pathways in subpleural regions (Suki and Bates, 2011; Mariano et al., 2022).

On imaging, quantitative CT (qCT) has become central to emphysema phenotyping, with well-validated density-mask thresholds (e.g., -950 HU) to estimate low-attenuation areas and emphysema burden (Gevenois et al., 1995; Lynch et al., 2015). Beyond density, 3D reconstruction and segmentation (e.g., Mimics, Geomagic) enable subject-specific geometry for mechanistic modeling and surgical planning (Tawhai et al., 2004).

Finite element (FE) studies from acinar to whole-lung scales show that geometry, heterogeneity, and boundary conditions profoundly shape local stress and strain. At the micro-scale, realistic alveolar geometries bear fully 3D stresses; stress amplifies near collapsed/edematous regions (Gefen et al., 1999; Sarabia-Vallejos et al., 2019; Chen et al., 2014). At the organ scale, heterogeneity and regional mechanics modulate ventilation patterns and strain (Tawhai et al., 2004; Avilés-Rojas and Hurtado, 2022). Most relevant here, biomechanical analyses of the thorax and pleura indicate greater mechanical stress at the apices, providing a mechanistic rationale for the apical predominance of blebs, bullae, and spontaneous pneumothorax (West, 1971; Mead et al., 1970; Casha et al., 2014).

Despite clinical significance, there are few datasets pairing ex vivo mechanical properties of human bullae with subject-specific FE simulations that predict stress localization and rupture risk under realistic loading. We therefore (i) measured nonlinear stress–strain behavior and rupture strength of resected human bulla and parenchyma specimens and fitted hyperelastic models, and (ii) constructed CT-derived geometry-specific FE models of 12 bullae from 10 surgical patients to analyze peak stresses and their anatomic localization under high-pressure loading. Our overarching aim was to place individualized stress maps in the context of ex vivo material failure to clarify where and how bullae fail.

## 2 Materials and methods

**Figure 1:**
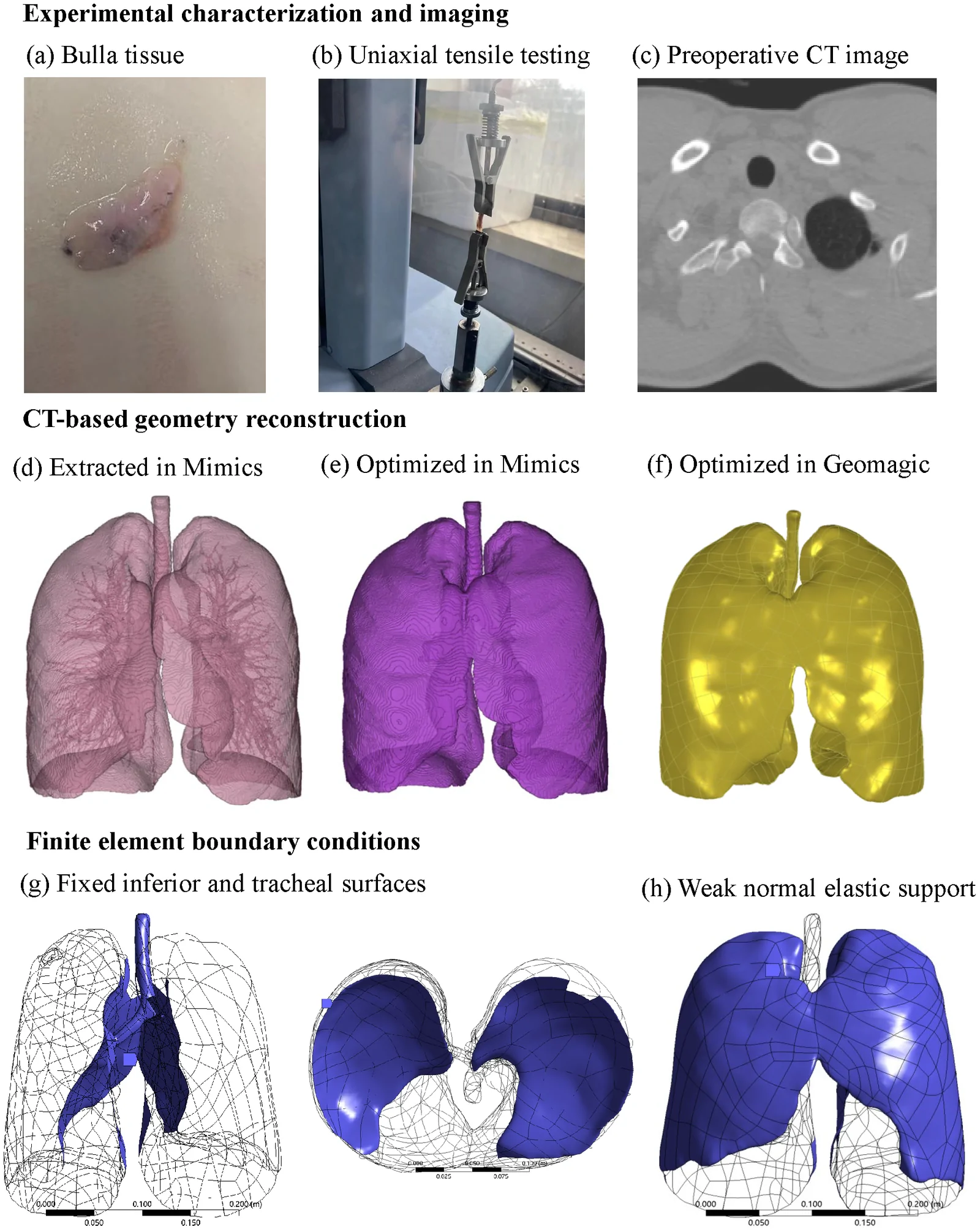
Experimental characterization, CT-based geometry reconstruction, and finite element boundary conditions. **(a)** Surgically resected bulla tissue. **(b)** Uniaxial tensile testing of the tissue specimen. **(c)** Preoperative CT image. **(d)** Initial lung geometry extracted in Mimics. **(e)** Lung geometry after optimization in Mimics. **(f)** Surface geometry after further optimization in Geomagic. **(g)** Fixed supports applied to the inferior lung and tracheal surfaces. **(h)** Weak normal elastic support applied to the lateral external lung surfaces.

The overall experimental, imaging, geometry-reconstruction, and finite element analysis workflow is summarized in **Figure 1**.

### 2.1 Human tissue collection and specimen preparation

Surgically resected bullae and adjoining parenchyma were placed in PBS in sterile centrifuge tubes and transported same-day to the biomechanics lab. Rectangular specimens were cut under a stereomicroscope; grip-to-grip distance, width, and central thickness were measured by calipers immediately before testing. Individual specimen dimensions are provided in **Supplementary Table 1**.

### 2.2 Uniaxial tensile tests

Uniaxial tensile tests were performed at room temperature using a 50 N *in-situ* uniaxial tester (IBTC-50-C-01; KAIR Co.) at a displacement rate of 0.04 mm/s until failure. Before testing, the initial specimen width ( $w_{0}$), central thickness ( $t_{0}$), and initial grip-to grip distance ( $L_{0}$) were measured. Axial load (F) and grip-to-grip displacement (ΔL) were recorded continuously.

Engineering stress and strain were calculated as,


$$\sigma_{eng}=F/(w_{0}t_{0})$$

and,


$$\epsilon_{eng}=\Delta L/L_{0}$$

respectively. No obvious specimen slippage was observed during testing or evident from the recorded load–displacement curves. Because strain was calculated from grip-to-grip displacement, no independent optical measurement of local gauge-region strain was performed.

Rupture location was visually classified as central-gauge or grip-adjacent. Ultimate tensile strength was defined as the maximum engineering stress. Only specimens that ruptured within the central gauge region were included in the UTS analysis, comprising 15 of 39 bulla specimens and 14 of 34 parenchyma specimens. Specimens that ruptured near the grips were excluded from the UTS analysis to reduce the influence of grip-associated failure.

**Figure 2:**
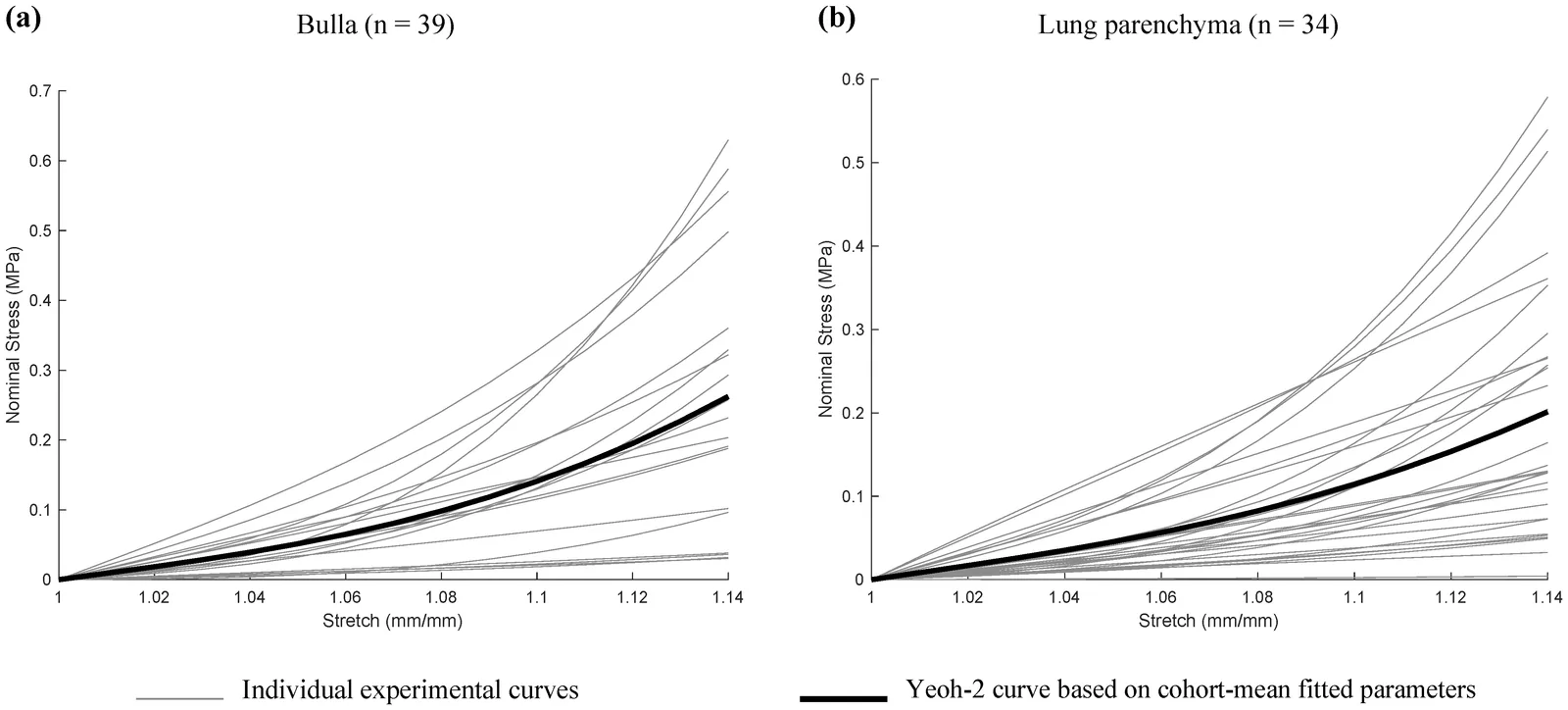
Uniaxial tensile responses and second-order Yeoh representations of bulla and lung parenchyma tissues. **(a)** Bulla tissue (n=39); **(b)** lung parenchyma (n=34).

We evaluated Neo-Hookean, Mooney–Rivlin, and Yeoh hyperelastic models (Yeoh, 1993; Mooney, 1940; Rivlin, 1948). Hyperelastic fitting was performed in MATLAB using the nonlinear least-squares function lsqcurvefit. Model performance was evaluated using the residual norm (resnorm), defined as the sum of squared residuals between the model-predicted and measured stress values. A lower resnorm therefore indicated a closer fit to the experimental data. Hyperelastic fitting was performed using the available loading data from all tested specimens, including specimens that subsequently ruptured near the grips, because no obvious slippage was observed before failure. Each specimen was fitted separately. The fitted parameters were subsequently averaged within each tissue group. The cohort-mean second-order Yeoh parameters were used to generate the thick constitutive curves shown in **Figure 2** and were assigned in the FE analyses.

The Yeoh hyperelastic model expresses the strain-energy density W as a polynomial of the first invariant of the (right) Cauchy–Green tensor, with an optional volumetric penalty for compressibility:


$$W=\sum_{i=1}^{n}C_{i}{\left(I_{1}-3\right)}^{i}+\sum_{k=1}^{n}\frac{1}{D_{k}}{\left(J-1\right)}^{2k}$$

Here $C_{i}$ are material constants that govern the deviatoric (shape-changing) response; $I_{1}=\lambda_{1}^{2}+\lambda_{2}^{2}+\lambda_{3}^{2}$ is the first invariant in terms of the principal stretches ${\text{λ}}_{1}$, ${\text{λ}}_{2}$, ${\text{λ}}_{3}$; $J={\text{λ}}_{1}{\text{λ}}_{2}{\text{λ}}_{3}$ is the volume ratio, and the parameters $D_{k}$ control compressibility (for an incompressible solid, J=1 and the volumetric term is omitted). In uniaxial tension of an incompressible material, ${\text{λ}}_{1}=\text{λ}$ and ${\text{λ}}_{2}={\text{λ}}_{3}={\text{λ}}^{-1/2}$, so $I_{1}={\text{λ}}^{2}+2{\text{λ}}^{-1}$. The commonly used second-order Yeoh model sets N = 2 (i.e., includes ( $I_{1}-3$) and ${\left(I_{1}-3\right)}^{2}$ terms), which is often sufficient to capture the pronounced strain-stiffening of soft tissues over large deformations.

### 2.3 3D digital reconstruction of the lung and bullae

Preoperative high-resolution volumetric chest CT datasets from the surgical candidates were used for three-dimensional reconstruction. Each dataset consisted of a 512 × 512 matrix with an in-plane pixel spacing of 0.854 × 0.854 mm and a slice thickness of 1.0 mm.

The lung regions were initially identified in Mimics using the Segment Airway and Segment Lung tools. The automatically generated masks were then manually corrected slice by slice using the Draw and Erase functions to restore omitted lung regions, remove vessels, spikes, and isolated noise, and delineate the bullae.

Three-dimensional lung and bulla models were generated using voxel-based reconstruction. Surface smoothing was performed in Mimics using a smoothing factor of 0.4 and five iterations. The models were exported as STL files and further processed in Geomagic Studio. The Mesh Doctor tool was used to repair mesh defects, residual spikes and outliers were manually removed, and the triangular meshes were converted into fitted NURBS surfaces through exact surfacing, automatic surface generation, surface adjustment, and surface fitting. Watertight geometries were subsequently exported in STEP format for finite element preprocessing.

### 2.4 Finite element analysis

Finite element analyses were performed in ANSYS Mechanical using the Static Structural module, with the large-deflection option enabled. The bulla was represented as a midsurface shell and discretized using SHELL181 elements with a centered thickness offset. The surrounding lung parenchyma was represented as a three-dimensional solid and discretized using SOLID187 tetrahedral elements. Bonded contact was prescribed between the bulla shell and the adjacent lung parenchyma.

The uniform baseline bulla-wall thickness was set to 0.5 mm based on direct measurements of the 39 bulla specimens obtained from 17 patients. The measured thickness was 0.468 ± 0.191 mm, with a median of 0.420 mm and a range of 0.190–0.960 mm.

Both the bulla wall and lung parenchyma were represented using second-order Yeoh hyperelastic models. For the bulla wall, the material parameters were C_1_ = 0.1525 MPa, C_2_ = 1.8660 MPa, D_1_ = 0.01 MPa^-1^, and D_2_ = 0. 1 MPa^-1^. For the lung parenchyma, the corresponding parameters were C_1_ = 0.1411 MPa, C_2_ = 1.2093 MPa, D_1_ = 0.01 MPa^-1^, and D_2_ = 0.1 MPa^-1^. The same tissue-specific cohort-level material parameters were assigned to all 12 geometries. The selected volumetric parameters represented a nearly incompressible material response. Their influence was evaluated in a separate compressibility sensitivity analysis.

Based on the mesh-convergence analysis, the final mesh used a target in-plane element size of 0.8 mm for the bulla shell. A spherical refinement region with a radius of 50 mm was defined around each bulla, within which the target lung-parenchyma element size was 2 mm. The target element size in the remaining remote lung parenchyma was 20 mm.

A uniform pressure of 110 cmH_2_O (10.787 kPa) was applied to the complete bulla shell in the direction producing outward expansion. This value was selected by rounding the upper airway-pressure level of 10.7 kPa (80 mmHg) reported for positive-pressure breathing during G protection (Glaser et al., 2017). The pressure was used as a conservative upper-bound stress-test load and was not interpreted as a direct measurement of the transmural pressure acting on a bulla in flight.

Fixed supports were applied to the inferior lung surfaces and the tracheal surface to prevent rigid-body motion. A weak normal elastic support with a foundation stiffness of 50 N·m^−3^ was applied to the lateral external surfaces of the lung. This support was used as a phenomenological weak restraint and was not interpreted as an experimentally calibrated chest-wall stiffness.

### 2.5 Output measures and descriptive statistics of FE models

Equivalent von Mises stress and maximum principal stress were evaluated exclusively over the bulla shell using the Top/Bottom shell result position with nodal averaging enabled. These settings were kept identical across all models and sensitivity analyses. All valid nodal stress values were sorted in ascending order, and the 95th- and 99th-percentile stresses were calculated using linear interpolation at p(N−1), where p is the specified percentile and N is the number of valid nodal values. Each nodal value was assigned equal weight, and no surface-area weighting was applied. Absolute maximum stresses were also recorded as secondary measures because of their greater sensitivity to local geometric irregularities and mesh refinement.

We reconstructed 12 bullae from 10 subjects (two subjects with two bullae each). For each bulla, the bulla-wall surface area, the 95th percentile, 99th percentile, and absolute maximum of equivalent stress, and the corresponding maximum-principal-stress measures were recorded. Descriptive statistics (median, IQR, range) were computed across cases; because the models represent different subjects (with two having multiple bullae), we treated each bulla as a case to summarize morphology and stress.

### 2.6 Mesh-convergence, wall-thickness sensitivity analyses, and compressibility sensitivity analyses

Mesh-convergence analyses were performed in three representative bulla models with distinct geometries and baseline stress levels. A baseline mesh and four progressively refined meshes were evaluated. The baseline target element sizes were 2.0 mm for the bulla shell and 5.0 mm for the lung parenchyma within the 50-mm spherical refinement region. The element sizes were subsequently divided by refinement factors of 1.5, 2.0, 2.5, and 3.0, corresponding to bulla-shell element sizes of 1.33, 1.00, 0.80, and 0.67 mm, respectively, and local lung-parenchyma element sizes of 3.33, 2.50, 2.00, and 1.67 mm, respectively. The target element size in the remote lung parenchyma was maintained at 20 mm. Mesh convergence was evaluated using the P95, P99, and absolute maximum values of equivalent von Mises stress and maximum principal stress. The 2.5-fold refined mesh was selected for the final analyses based primarily on stabilization of the P95 stress measures across the three representative models.

Wall-thickness sensitivity was evaluated in the same three representative models using uniform bulla-wall thicknesses of 0.3, 0.5, and 0.7 mm. All other material parameters, contact definitions, mesh settings, pressure loads, and boundary conditions were kept unchanged. Wall-thickness sensitivity was evaluated using the P95, P99, and absolute maximum values of equivalent von Mises stress and maximum principal stress.

Parenchymal-compressibility sensitivity was evaluated in the same three representative models by varying the first volumetric parameter D_1_ from the baseline value of 0.01 MPa^-1^ to 0.733 and 1.519 MPa^-1^. These values corresponded to equivalent initial Poisson’s ratios of approximately 0.499, 0.450, and 0.400, respectively. D_2_ was maintained at 0.1 MPa^-1^. The deviatoric material parameters, all bulla-wall material parameters, wall thickness, mesh settings, loading, contact definitions, and boundary conditions were unchanged. Sensitivity was evaluated using the P95, P99, and absolute maximum values of equivalent von Mises stress and maximum principal stress.

### 2.7 Descriptive statistical analysis

UTS data from specimens that ruptured within the central gauge region were summarized using the sample size, mean and standard deviation, median, interquartile range, and full range.

Twelve bullae were reconstructed from 10 subjects, with two subjects contributing two bullae each. Each bulla was treated as an individual case for descriptive summarization. Bulla-wall surface area and finite element stress measures were summarized using the median, interquartile range, and full range. Because two subjects contributed more than one bulla, no inferential analyses assuming complete independence among all 12 cases were performed.

## 3 Results

### 3.1 Hyperelastic material behavior

Both tissues exhibited a classic toe region followed by marked stiffening. Among the three candidate constitutive models, the second-order Yeoh model provided the lowest mean residual norm across the bulla and parenchyma datasets. The mean resnorm was 3.703 ± 13.320 for the Yeoh model, compared with 3.737 ± 16.973 for the Mooney–Rivlin model and 59.998 ± 183.997 for the Neo-Hookean model. The second-order Yeoh model was therefore selected for the finite element analyses because it provided the best overall representation of the nonlinear experimental response. In Yeoh-2 fits, the material parameters (mean) were: Parenchyma C_1_ = 0.1411 MPa, C_2_ = 1.2093 MPa; Bulla C_1_ = 0.1525 MPa, C_2_ = 1.866 MPa. Cohort-mean Yeoh curves are shown in **Figure 2**.

The slightly higher C_1_ in bullae indicates a somewhat stiffer initial response at small strains, while the notably higher C_2_ reflects stronger strain-stiffening at large deformations. Together, these suggest that compared with parenchyma, bulla tissue resists stretch more strongly as deformation increases, consistent with collagen-dominated load bearing once the elastin network is exhausted (Andrikakou et al., 2016; Polio et al., 2018; Suki and Bates, 2011).

### 3.2 Ultimate tensile strength

Among specimens that ruptured within the central gauge region, the ultimate tensile strength of bulla tissue (n=15) was 2.318±1.541 MPa, with a median of 2.330 MPa, an interquartile range of 1.226-3.027 MPa, and a range of 0.462-5.066 MPa. For lung parenchyma specimens (n=14), the corresponding ultimate tensile strength was 0.390±0.210 MPa, with a median of 0.348 MPa, an interquartile range of 0.254-0.446 MPa, and a range of 0.153-0.813 MPa. Individual values and descriptive statistics are provided in **Table 1**.

**Table 1:** Individual failure stresses and descriptive statistics of lung bulla and parenchyma specimens.

Bulla specimen no.	Bulla UTS (MPa)	Parenchyma specimen No.	Parenchyma UTS (MPa)
1	5.066	1	0.336
2	4.629	2	0.467
3	3.535	3	0.813
4	1.014	4	0.593
5	1.437	5	0.360
6	2.445	6	0.383
7	0.504	7	0.161
8	1.615	8	0.153
9	2.520	9	0.375
10	2.330	10	0.306
11	1.514	11	0.299
12	0.649	12	0.784
13	2.342	13	0.239
14	4.704	14	0.187
15	0.462		
n	15	n	14
Mean ± SD	2.318 ± 1.541	Mean ± SD	0.390 ± 0.210
Median	2.330	Median	0.348
IQR	1.226-3.027	IQR	0.254-0.446
Range	0.462-5.066	Range	0.153-0.813

UTS, ultimate tensile strength; SD, standard deviation; IQR, interquartile range. Only specimens that ruptured within the central gauge region were included in the UTS analysis.

### 3.3 CT-derived geometry-specific finite element results

Final-mesh results were obtained for all 12 CT-derived bulla models using the baseline uniform wall thickness of 0.5 mm. Bulla-wall surface area ranged from 162.7 to 1089.3 mm², with a median of 393.9 mm². The 95th-percentile equivalent von Mises stress ranged from 50.3 to 515.8 kPa, with a median of 106.8 kPa, whereas the 95th-percentile maximum principal stress ranged from 55.6 to 303.8 kPa, with a median of 121.1 kPa. Complete case-level P95, P99, and absolute-maximum stress measures are provided in **Table 2**.

**Table 2:** Case-level geometric and finite element stress measures for CT-derived lung bulla models.

Case no.	Bulla-wall surface area (mm²)	Equivalent von Mises stress (kPa)			Maximum principal stress (kPa)		
		P95	P99	Maximum	P95	P99	Maximum
1	513.5	103.3	145.1	189.0	115.3	159.8	198.1
2	166.8	66.1	87.6	109.2	74.0	97.5	114.6
3	1089.3	157.8	207.0	247.3	173.7	226.1	279.8
4	1074.2	173.5	246.0	314.4	199.1	283.1	354.3
5	354.3	59.8	118.7	287.6	68.9	137.0	331.2
6	319.9	80.8	106.9	121.2	88.0	113.9	128.0
7	332.5	71.4	97.5	161.4	81.6	109.6	183.0
8	412.1	110.2	174.4	295.1	127.0	201.2	345.7
9	1082.9	206.5	287.5	603.7	236.9	331.7	693.8
10	162.7	50.3	67.1	92.5	55.6	72.9	95.2
11	512.0	315.9	487.9	1060.1	188.3	290.7	617.6
12	375.7	515.8	775.2	1099.9	303.8	468.4	671.1
n	12	12	12	12	12	12	12
Median	393.9	106.8	159.7	267.5	121.1	180.5	305.5
IQR	329.4-653.6	70.0-181.7	104.5-256.3	151.4-386.7	79.7-191.0	112.8-285.0	169.2-420.1
Range	162.7-1089.3	50.3-515.8	67.1-775.2	92.5-1099.9	55.6-303.8	72.9-468.4	95.2-693.8

P95 and P99 denote the 95th and 99th percentiles, respectively. Percentiles were calculated from valid nodal values using linear interpolation at p(N−1), with equal node weighting and without surface-area weighting. Results correspond to the final 2.5-fold refined mesh, a uniform bulla-wall thickness of 0.5 mm, the Top/Bottom shell result position, and nodal averaging enabled.

**Figure 3:**
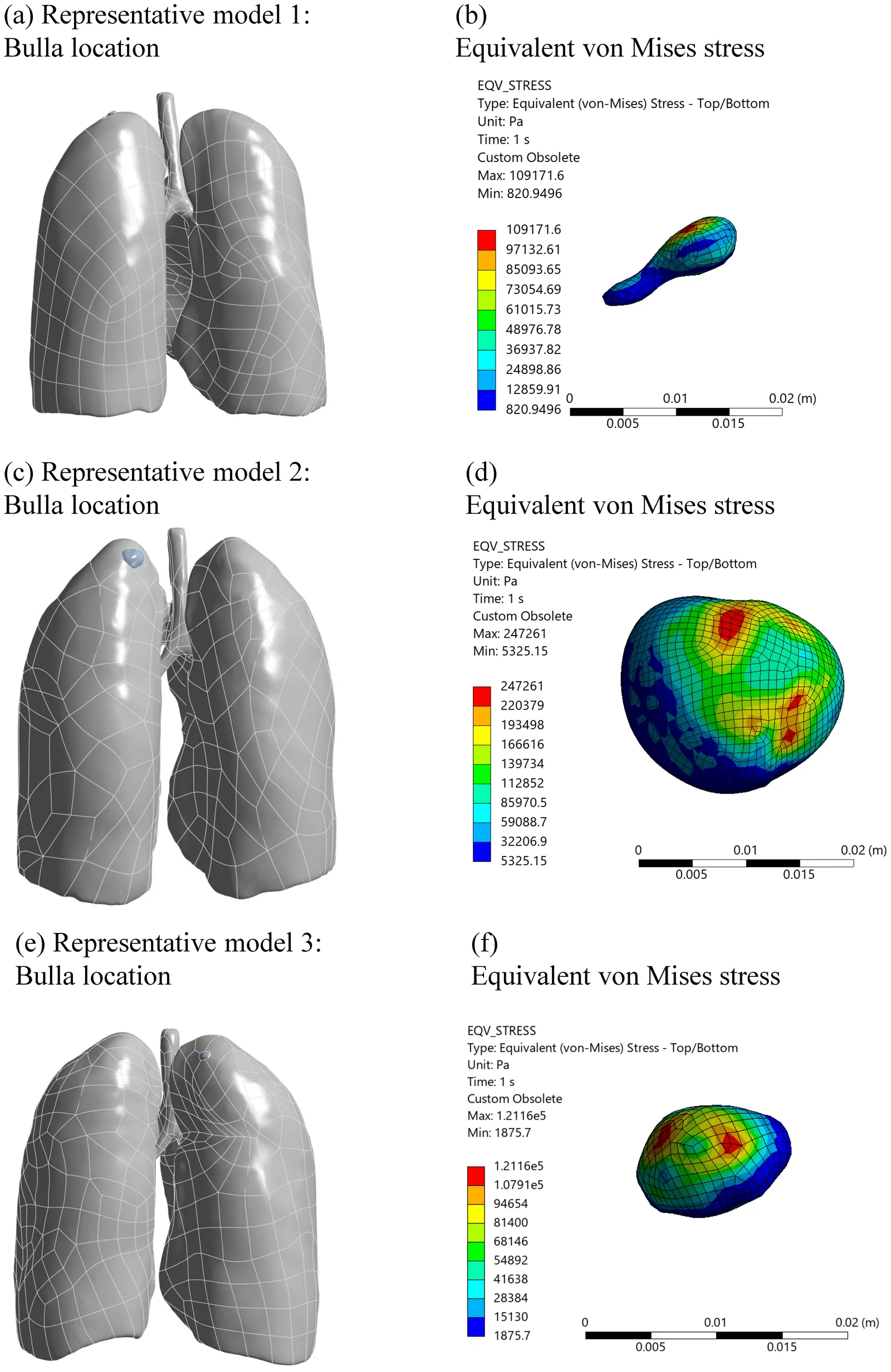
Locations and equivalent von Mises stress distributions of three representative CT-derived geometry-specific bulla models under a pressure load of 110 cmH_2_O. Panels **(a)**, **(c)**, and **(e)** show the reconstructed lung geometries and the locations of the bullae in representative models 1–3, respectively. Panels **(b)**, **(d)**, and **(f)** show the corresponding equivalent von Mises stress distributions in the bulla shells. Representative models 1–3 correspond to Cases 2, 3, and 6, respectively, and were also used in the mesh-convergence, wall-thickness, and parenchymal-compressibility sensitivity analyses. Individual color scales are used to visualize the stress distribution within each model; quantitative comparisons among models are provided in **Table 2**.

Representative stress distributions are shown in **Figure 3**. Elevated stresses were mainly localized to the bulla vertex (apex), i.e., the most remote convex tip of the bulla protruding toward the pleural surface.

The FE stresses were compared descriptively with the experimentally measured bulla UTS distribution. The highest P95 equivalent von Mises stress was 515.8 kPa, which exceeded the minimum measured bulla UTS of 462 kPa but remained below the mean bulla UTS of 2.318 MPa. Because this comparison involved multiaxial FE loading, cohort-level material properties, a uniform wall-thickness assumption, and non-paired experimental and computational datasets, it was not interpreted as a validated rupture criterion.

### 3.4 Mesh-convergence analysis

**Figure 4:**
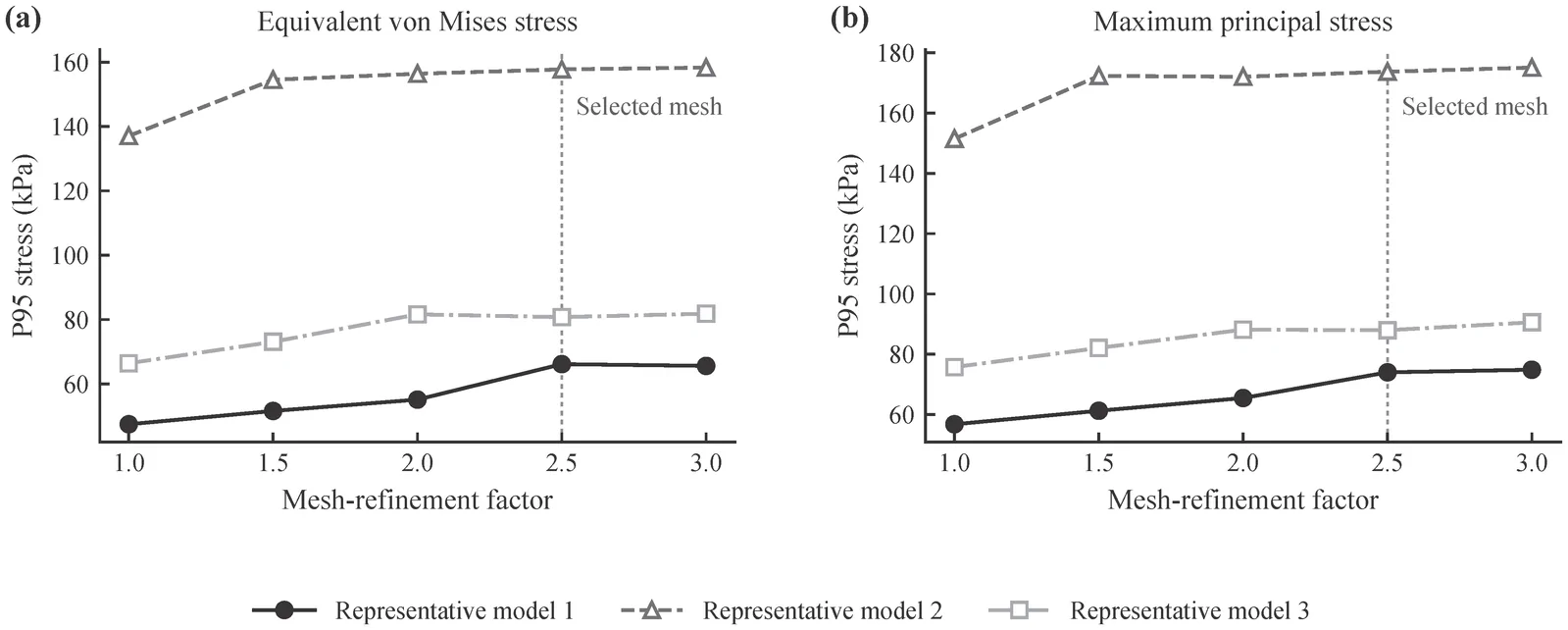
Mesh convergence of the primary finite element stress measures in three representative bulla models. Changes in **(a)** the 95th-percentile equivalent von Mises stress and **(b)** the 95th-percentile maximum principal stress are shown across five mesh-refinement factors. The vertical dashed line indicates the 2.5-fold refined mesh selected for the final analyses. The three representative models were selected to include different bulla geometries and baseline stress levels.

Between the 2.5-fold and 3.0-fold refined meshes, changes in the 95th-percentile equivalent von Mises and maximum principal stresses were no greater than 3.0% across the three representative models. The P99 and absolute-maximum measures were more mesh sensitive, with changes of up to 8.2% and 10.6%, respectively. The 2.5-fold refined mesh was therefore selected for the final analyses based primarily on stabilization of the P95 measures (**Figure 4**).

### 3.5 Wall-thickness sensitivity

**Figure 5:**
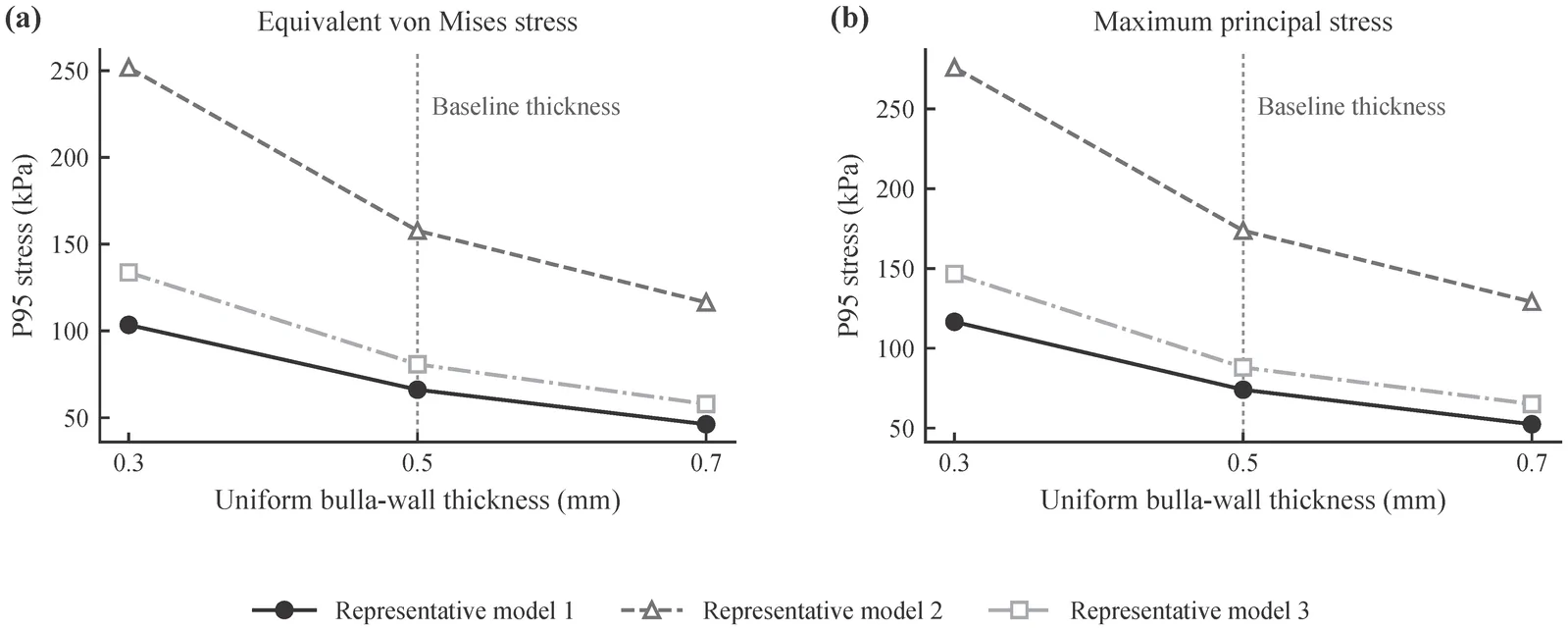
Sensitivity of the primary finite element stress measures to the assumed uniform bulla-wall thickness. Changes in **(a)** the 95th-percentile equivalent von Mises stress and **(b)** the 95th-percentile maximum principal stress are shown for three representative bulla models at wall thicknesses of 0.3, 0.5, and 0.7 mm. The vertical dashed line indicates the baseline wall thickness of 0.5 mm used in the final analyses. The analysis quantifies the dependence of the predicted stresses on the uniform wall-thickness assumption and does not represent patient-specific wall-thickness measurements.

Wall thickness substantially affected the computed stresses in all three representative models. Relative to the 0.5-mm baseline, reducing the wall thickness to 0.3 mm increased P95 equivalent von Mises stress by 56.5%-65.6% and P95 maximum principal stress by 57.5%-66.6%. Increasing the wall thickness to 0.7 mm reduced the corresponding stresses by 26.2%-30.1% and 25.7%-29.3%, respectively (**Figure 5**). Thus, the predicted stress magnitude was strongly dependent on the assumed bulla-wall thickness.

### 3.6 Parenchymal-compressibility sensitivity

Varying the parenchymal D_1_ parameter from 0.010 to 0.733 and 1.519 MPa^-1^ produced only minor changes in bulla-wall stress. Across the three representative models, changes in P95 equivalent von Mises stress ranged from -0.10% to 0.25%, whereas changes in P95 maximum principal stress ranged from 0.04% to 0.52%. The absolute change in every evaluated P95, P99, and maximum stress measure remained below 0.53%, indicating that the predicted bulla-wall stresses were insensitive to the tested range of parenchymal compressibility (**Supplementary Table 2**).

## 4 Discussion

This study integrates ex vivo hyperelastic characterization of human bulla and parenchyma tissues with CT-derived geometry-specific finite element modeling to examine where bullae concentrate stress. The tensile tests demonstrate that bulla walls are mechanically stronger than parenchyma on average, yet they also exhibit substantial inter-specimen variability, with some specimens failing at relatively low stress. The finite element simulations indicate that while most bullae experience peak stresses below the average rupture threshold under high internal pressure, some models exhibited local stresses that exceeded the minimum measured bulla UTS. This observation underscores the role of anatomy, particularly curvature at the apex or vertex, in predisposing bullae to rupture (West, 1971; Mead et al., 1970; Casha et al., 2014).

The consistent localization of stress at the bulla vertex is mechanically intuitive. In thin-walled pressurized shells, local curvature accentuates membrane tension. At the same time, the vertex is less tethered by adjacent parenchyma than equatorial regions, which limits load sharing and increases local stress. Classical studies of lung mechanics have long demonstrated that regional tethering and pleural coupling influence local strain distribution (Casha et al., 2014; West, 1971; Mead et al., 1970). More recent finite element studies at the alveolar level confirm that geometric heterogeneity can magnify local stress and strain (Gefen et al., 1999; Sarabia-Vallejos et al., 2019). The present findings therefore bridge tissue-level mechanics and organ-level simulations, highlighting the apex as the mechanically most vulnerable site.

Negative pleural pressure is more pronounced at the lung apices, and resting alveoli in these regions are larger and less compliant. This anatomical gradient predisposes the apices to higher mechanical stresses during strain excursions (West, 1971; Mead et al., 1970). Thoracic finite element analyses have also shown higher stress distributions in apical regions, which provides a mechanistic explanation for the clinical observation that spontaneous pneumothorax most often originates from apical blebs or bullae (Hallifax, 2022; Park et al., 2019; Casha et al., 2014).

The material modeling results are also informative. Among the candidate constitutive laws, the Yeoh second-order model provided the best fit across the strain range, capturing both the initial compliance and subsequent strain stiffening. The slightly higher C_1_value in bullae compared with parenchyma reflects a modestly stiffer initial response, while the more substantial increase in C_2_ reflects stronger nonlinear stiffening at large deformations. This behavior is consistent with the progressive recruitment of collagen fibers as the elastin network is stretched, a mechanism that has been emphasized in prior studies of lung tissue mechanics (Andrikakou et al., 2016; Polio et al., 2018; Suki and Bates, 2011). Although Ogden models can also capture strong nonlinearities, they require more parameters and can be difficult to calibrate reliably from uniaxial data alone (Yeoh, 1993; Rivlin, 1948). The Yeoh formulation strikes a pragmatic balance, which makes it suitable for incorporating into finite element simulations informed by experimental data.

The finding that bulla tissue appeared stiffer than parenchyma in uniaxial tests is consistent with the structural differences between the two tissues. Normal lung parenchyma consists of a highly compliant network of alveoli with thin septa and large air content, resulting in low bulk stiffness. In contrast, bullae arise from progressive alveolar wall destruction and remodeling, leaving behind compressed and often fibrotic tissue layers that define the cavity wall. These remodeled walls may also incorporate contributions from adjacent pleura or fibrotic adhesions, thereby increasing local tissue density and mechanical resistance. Such structural consolidation explains why bulla tissue can exhibit greater stiffness and higher ultimate tensile strength than the surrounding parenchyma.

Although bulla tissue demonstrated higher stiffness and greater ultimate tensile strength than parenchyma in our uniaxial tests, this does not guarantee resistance to rupture *in vivo*. The propensity for rupture is strongly influenced by macroscopic geometry as well as material strength. According to Laplace’s law for thin-walled spheres, wall stress is proportional to the product of internal pressure and radius, and inversely proportional to wall thickness. Thus, at a given pressure and thickness, larger bullae generate substantially higher wall stresses than smaller alveoli. These amplified stresses may approach or exceed the rupture threshold despite the relatively strong material properties of bulla tissue. This geometric amplification provides a mechanistic explanation for why bullae can rupture under physiological pressures even when adjacent parenchyma remains intact.

Our results align with clinical imaging and outcome studies. High-resolution CT is the gold standard for detecting blebs and bullae, and quantitative CT has established metrics for emphysema burden (Gevenois et al., 1995; Lynch et al., 2015). Several studies, however, have reported that the mere presence of apical blebs or bullae on CT does not always predict recurrence of pneumothorax, which has led to debate about their role (Noppen, 2010; Grundy et al., 2012). The present findings help resolve this tension by providing a mechanical rationale: small apical lesions may disproportionately elevate stress at their vertices, even if their volumes are modest, thereby creating focal sites at risk of rupture. Integrating CT-derived geometry-specific finite element modeling with CT morphology can therefore add mechanistic insight beyond conventional volumetric or density-based emphysema measures.

The choice of reporting the 95th-percentile stress rather than the absolute maximum deserves emphasis. Mesh artifacts and geometric singularities often inflate the stress in individual elements, which can misrepresent true physiological loading. Percentile-based reporting has been recommended as a more robust metric, reflecting stress in a small but finite region rather than at a single node (Sinclair et al., 2019; Knowles et al., 2022). This approach allowed us to compare cases fairly and to link computational outputs more directly with experimental rupture stresses. However, this approach may smooth out small but physiologically relevant stress peaks. In reality, even small focal regions of very high stress could act as rupture initiation sites, particularly if coincident with tissue defects. Thus, while percentile-based metrics improve robustness, they may underestimate true peak vulnerability. Future work should consider both percentile metrics and local maxima, together with mesh-refinement studies, to capture clinically meaningful stress hot-spots.

The computational models cannot account for pre-existing micro-lesions, tissue fractures, or localized degenerative changes in the extracellular matrix that may predispose a bulla to rupture. Collagen-network remodeling and elastin degradation may create local mechanical weak points and reduce tissue failure strength, although these microstructural abnormalities were not represented in the present models (Kononov et al., 2001; Ito et al., 2005). Integrating multiscale modeling approaches or incorporating histological data could help bridge this gap in future studies.

Several limitations of this study should be acknowledged. First, the applied pressure of 110 cmH_2_O represents an extreme condition chosen as a “stress test,” and while cough or Valsalva maneuvers can generate large transient pressures, future simulations should explore a broader physiological range. Second, we modeled bulla walls with a uniform thickness of 0.5 mm. Although this value was based on the mean thickness measured in 39 bulla specimens, it cannot represent the substantial between-specimen and spatial variation in wall thickness. The wall-thickness sensitivity analysis confirmed that this assumption strongly affected the predicted stress magnitude. If the actual wall is thinner than assumed, the same internal pressure would generate greater local stresses, potentially exceeding the measured ultimate tensile strength and precipitating rupture. Incorporating patient-specific wall thickness maps from future high-resolution imaging modalities could therefore improve predictive accuracy. Third, the constitutive models were calibrated solely from uniaxial tension, which may not fully capture multiaxial stress states in thin shells. Biaxial or inflation testing, coupled with inverse finite element fitting, would provide more robust anisotropic parameters. Fourth, the lateral elastic support was implemented as a phenomenological weak restraint rather than an experimentally calibrated representation of chest-wall or pleural mechanics. Because foundation-stiffness sensitivity was not evaluated, its influence on load transfer and predicted bulla-wall stress remains uncertain, and the reported results should therefore be interpreted within the specified boundary-condition set. In addition, strain was calculated from grip-to-grip displacement without independent optical measurement of local gauge-region deformation. Although no obvious gross slippage was observed, subtle progressive slippage and grip compliance cannot be excluded. The fitted parameters should therefore be interpreted as effective parameters for the tested configuration. Restricting the UTS analysis to central-gauge failures may also introduce selection bias by preferentially retaining more uniform or less locally damaged specimens. Finally, our dataset of twelve bullae from ten patients limits the generalizability of the results, although it demonstrates the feasibility of pairing ex vivo mechanics with *in vivo* imaging.

Despite these limitations, this study offers a new framework for understanding why bullae rupture where they do. By integrating ex vivo material testing with subject-specific finite element simulations, we show that the vertex or apex is mechanically the most vulnerable site, and that some bullae can reach failure-relevant stresses even when the mean tissue strength remains protective. This perspective provides a mechanistic explanation for the apical predilection of spontaneous pneumothorax and suggests that finite element–based risk stratification could augment clinical decision-making for bullectomy or surveillance in patients with bullous lung disease.

## Data Availability

The datasets presented in this article are not readily available because their use is restricted to non-commercial purposes. Requests to access the datasets for academic research, education, or public-welfare purposes should be directed to Yan Huang at huangyan0416@126.com.

## Supplementary material

Supplementary Table 1 Initial dimensions of bulla and lung parenchyma specimens used for uniaxial tensile testing. Panel **(A)** Lung bulla specimens. Panel **(B)** Lung parenchyma specimens.

Supplementary Table 2 Sensitivity of bulla-wall stress measures to the assumed parenchymal compressibility.
