# Supplementary material for: Biomechanical characterization of human lung bullae using uniaxial tensile tests and CT-derived geometry-specific finite element analysis
Source: Front Physiol. 2026 Aug 7;17:1818153. doi: 10.3389/fphys.2026.1818153 (PMC13494262; doi:10.3389/fphys.2026.1818153)
Supplement: Supplementary file 1 [file Table1.pdf]

Table S1. Initial dimensions of bulla and lung parenchyma specimens used for uniaxial tensile testing

Panel A. Lung bulla specimens

Specimen No.	Initial central thickness (mm)	Initial width (mm)	Initial grip-to-grip distance (mm)
1	0.49	6.42	10.63
2	0.63	5.01	6.91
3	0.69	5.05	12.08
4	0.65	8.17	8.83
5	0.52	2.33	12.20
6	0.94	7.05	11.92
7	0.69	6.05	11.78
8	0.37	6.19	7.74
9	0.54	6.23	9.11
10	0.51	7.04	9.29
11	0.47	10.31	11.60
12	0.96	5.64	6.04
13	0.88	5.47	8.35
14	0.25	3.34	11.81
15	0.19	2.94	13.32
16	0.78	3.17	8.83
17	0.35	2.63	5.53
18	0.33	2.74	7.77
19	0.32	2.88	8.21
20	0.54	4.18	8.78
21	0.33	3.43	15.43
22	0.59	6.34	7.69
23	0.30	1.76	9.33
24	0.37	3.01	6.35
25	0.33	4.76	11.08
26	0.41	3.86	10.72
27	0.44	2.55	5.99
28	0.22	3.27	5.87
29	0.39	3.50	4.63
30	0.46	2.40	3.69
31	0.49	4.34	4.45
32	0.34	3.71	4.92
33	0.36	3.03	3.96
34	0.42	4.28	0.91
35	0.40	4.01	3.38
36	0.23	5.25	5.82
37	0.42	2.52	3.22
38	0.28	3.00	3.66
39	0.39	2.81	6.84

Specimen No.	Initial central thickness (mm)	Initial width (mm)	Initial grip-to-grip distance (mm)
n	39	39	39
Mean ± SD	0.468 ± 0.191	4.376 ± 1.864	7.915 ± 3.307
Median	0.420	3.860	7.770
IQR	0.335-0.540	2.970-5.555	5.675-10.675
Range	0.190-0.960	1.760-10.310	0.910-15.430

Panel B. Lung parenchyma specimens

Specimen No.	Initial central thickness (mm)	Initial width (mm)	Initial grip-to-grip distance (mm)
1	1.60	4.43	7.82
2	1.07	8.02	13.17
3	0.81	5.91	7.54
4	0.87	7.93	9.93
5	0.66	2.35	11.69
6	0.80	5.21	8.69
7	0.68	3.25	9.04
8	0.46	3.46	11.59
9	1.02	7.04	8.70
10	1.22	8.16	9.31
11	0.87	8.63	9.07
12	1.56	6.00	9.71
13	0.71	5.72	10.49
14	0.83	3.37	8.75
15	0.91	2.77	10.37
16	0.90	6.51	8.88
17	1.41	6.30	12.04
18	0.76	5.02	7.35
19	0.61	6.75	6.43
20	0.80	4.76	7.72
21	0.87	8.51	5.77
22	1.04	6.66	10.45
23	1.42	7.43	9.29
24	0.90	6.09	9.31
25	1.17	6.96	5.10
26	0.84	5.70	10.24
27	1.09	6.71	12.87
28	0.67	7.62	8.73
29	1.01	8.13	5.98
30	1.37	8.49	7.89
31	1.51	6.36	7.60
32	0.87	5.81	6.05
33	0.76	4.60	7.69

**Specimen No.**	**Initial central thickness (mm)**	**Initial width (mm)**	**Initial grip-to-grip distance (mm)**
34	0.83	6.36	11.49
n	34	34	34
Mean ± SD	0.968 ± 0.288	6.089 ± 1.718	9.022 ± 2.018
Median	0.870	6.330	8.960
IQR	0.800-1.085	5.068-7.333	7.698-10.338
Range	0.460-1.600	2.350-8.630	5.100-13.170
