# Supplementary material for: Biomechanical characterization of human lung bullae using uniaxial tensile tests and CT-derived geometry-specific finite element analysis
Source: Front Physiol. 2026 Aug 7;17:1818153. doi: 10.3389/fphys.2026.1818153 (PMC13494262; doi:10.3389/fphys.2026.1818153)
Supplement: Supplementary file 2 [file Table2.pdf]

**Table S2.** Sensitivity of bulla-wall stress measures to the assumed parenchymal compressibility

| Model | Parenchymal properties | | Equivalent von Mises stress | | | | | | Maximum principal stress | | | | | |
|---|---|---|---|---|---|---|---|---|---|---|---|---|---|---|
| | $D_1$ | $\nu_0$ | P95 | Δ P95 | P99 | Δ P99 | Max | Δ Max | P95 | Δ P95 | P99 | Δ P99 | Max | Δ Max |
| | 0.010 | 0.499 | 66.14 | 0.00 | 87.60 | 0.00 | 109.17 | 0.00 | 74.01 | 0.00 | 97.45 | 0.00 | 114.57 | 0.00 |
| 1 | 0.733 | 0.450 | 66.13 | -0.02 | 87.58 | -0.02 | 109.20 | +0.03 | 74.05 | +0.05 | 97.83 | +0.39 | 114.65 | +0.07 |
| | 1.519 | 0.400 | 66.21 | +0.11 | 87.57 | -0.03 | 109.19 | +0.02 | 74.04 | +0.04 | 97.87 | +0.43 | 114.65 | +0.07 |
| | 0.010 | 0.499 | 157.78 | 0.00 | 207.03 | 0.00 | 247.26 | 0.00 | 173.72 | 0.00 | 226.13 | 0.00 | 279.78 | 0.00 |
| 2 | 0.733 | 0.450 | 158.01 | +0.15 | 207.16 | +0.06 | 247.55 | +0.12 | 174.16 | +0.25 | 226.19 | +0.03 | 279.33 | -0.16 |
| | 1.519 | 0.400 | 158.17 | +0.25 | 207.26 | +0.11 | 247.70 | +0.18 | 174.63 | +0.52 | 226.23 | +0.04 | 279.51 | -0.10 |
| | 0.010 | 0.499 | 80.76 | 0.00 | 106.90 | 0.00 | 121.16 | 0.00 | 87.98 | 0.00 | 113.91 | 0.00 | 128.04 | 0.00 |
| 3 | 0.733 | 0.450 | 80.71 | -0.06 | 106.96 | +0.06 | 121.11 | -0.04 | 88.27 | +0.33 | 114.08 | +0.15 | 128.04 | 0.00 |
| | 1.519 | 0.400 | 80.68 | -0.10 | 106.96 | +0.06 | 121.10 | -0.05 | 88.26 | +0.32 | 114.10 | +0.17 | 128.04 | 0.00 |

$D_1$ denotes the first volumetric parameter of the second-order Yeoh model and is reported in $MPa^{-1}$. The baseline $D_1$ value was 0.010 $MPa^{-1}$. $\nu_0$ denotes the equivalent initial Poisson's ratio calculated from the initial shear and bulk moduli and does not represent an experimentally measured constant Poisson's ratio over finite deformation. $D_2$ was maintained at 0.100 $MPa^{-1}$ in all analyses. Stress values are reported in kPa, and Δ values indicate percentage changes relative to the corresponding baseline result within each representative model. All bulla-wall material parameters, wall thickness, mesh settings, loading, contact definitions, and boundary conditions were unchanged.
